# Contemporaneous symptom networks of multidimensional symptom experiences in cancer survivors: A network analysis

**DOI:** 10.1002/cam4.4904

**Published:** 2022-06-01

**Authors:** Zheng Zhu, Yanling Sun, Yi Kuang, Xiaoyi Yuan, Haiyan Gu, Jing Zhu, Weijie Xing

**Affiliations:** ^1^ School of Nursing Fudan University Shanghai China; ^2^ Fudan University Centre for Evidence‐based Nursing: A Joanna Briggs Institute Centre of Excellence Fudan University Shanghai China; ^3^ School of Public Health Fudan University Shanghai China; ^4^ Department of Chronic Disease Prevention and Control Xuhui District Center for Disease Control and Prevention Shanghai China

**Keywords:** cancer, network analysis, survivorship, symptom network

## Abstract

**Background:**

Symptom networks can provide empirical evidence for the development of personalized and precise symptom management strategies. However, few studies have explored the symptom networks of multidimensional symptom experiences in cancer survivors. The objectives of this study were to generate symptom networks of multidimensional symptom experiences in cancer survivors and explore the centrality indices and density in these symptom networks

**Methods:**

Data from 1065 cancer survivors were obtained from the Shanghai CANcer Survivor (SCANS) Report. The MD Anderson Symptom Inventory was used to assess the prevalence and severity of 13 cancer‐related symptoms. We constructed contemporaneous networks with all 13 symptoms after controlling for covariates.

**Results:**

Distress (*r*_*s*_ = 9.18, *r*_*c*_ = 0.06), sadness (*r*_*s*_ = 9.05, *r*_*c*_ = 0.06), and lack of appetite (*r*_*s*_ = 9.04, *r*_*c*_ = 0.06) had the largest values for strength and closeness. The density of the “less than 5 years” network was significantly different from that of the “5–10 years” and “over 10 years” networks (*p* < 0.001). We found that while fatigue was the most severe symptom in cancer survivorship, the centrality of fatigue was lower than that of the majority of other symptoms.

**Conclusion:**

Our study demonstrates the need for the assessment of centrality indices and network density as an essential component of cancer care, especially for survivors with <5 years of survivorship. Future studies are warranted to develop dynamic symptom networks and trajectories of centrality indices in longitudinal data to explore causality among symptoms and markers of interventions.

## Graphical abstract

This study use data from 1065 cancer survivors from the Shanghai CANcer Survivor (SCANS) Report. We constructed contemporaneous networks with all 13 symptoms after controlling for covariates. We found that while fatigue was the most severe symptom in cancer survivorship, the centrality of fatigue was lower than that of the majority of other symptoms.

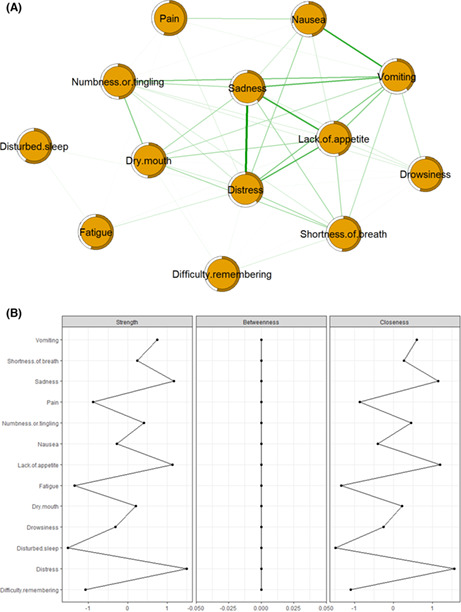

## 1 INTRODUCTION

Due to early detection, improved diagnostic procedures, and optimized treatment, the number of cancer survivors has gradually risen over the last decade. There have been an estimated 43.8 million cancer survivors in the last 5 years globally.^1^, ^2^ As survivors live longer, new challenges emerge. It has been widely reported that a number of cancer survivors experience high levels of symptom burden due to both treatments and the cancer itself.^3^, ^4^ Cancer survivors, especially survivors with breast, gynecological, prostate, and colon cancer, experienced 6–9 symptoms at the same time according to previous systematic reviews.^5^ Among all symptoms, depressive symptoms, pain, and fatigue were the most prevalent and severe symptoms across all cancer groups.^3^, ^5^, ^6^ Identifying and managing symptoms in cancer survivors are critical because high levels of symptom burden decrease individuals' physical and psychological function.^7^

Most studies have separately explored symptoms, which ignores the complex relationships among multiple symptoms. Previous studies have reported that cancer survivors are experiencing 6–9 symptoms at the same time.^5^, ^8^ Symptoms may have synergistic effects that worsen other symptoms and ultimately lead to low levels of quality of life.^9^ The mechanism cause of symptom deterioration and alleviation varied in different symptom combinations. The most common synergistic effects of cancer‐related symptoms were depression/fatigue/insomnia and lack of appetite/nausea/vomiting/diarrhea.^10^, ^11^ Understanding how multiple symptoms interact with each other is crucial to help healthcare providers handle targeted symptoms and prevent the occurrence of related symptoms at the same time. Over 20 types of cancer‐specific symptom checklists have been developed to face this challenge, such as the MD Anderson Symptom Inventory (MDASI), the Memorial Symptom Assessment Scale (MSAS), the Edmonton Symptom Assessment System (ESAS), and the Symptom Reporting Tool.^12^, ^13^, ^14^, ^15^

Identifying symptom clusters is a commonly used scientific approach for dimension reduction to simplify complex relationships among symptoms in real‐world clinical practice. Based on Dodd's definition, a “symptom cluster” refers to two or more concurrent symptoms that may or may not have the same etiology.^16^ Exploring symptom clusters is a widely used analytical paradigm. However, many reviews have noted that the combinations of symptoms in clusters may vary due to the selection of symptoms included in the analysis, the statistical methods used, and other covariates.^17^, ^18^ Whether the dimension reduction approach to exploring symptom clusters suits today's clinical practice with large amounts of big data continues to be debated.^19^

Categorizing symptoms provides only a broad picture of which cancer‐related symptoms share the same co‐occurring mechanisms, and how the symptoms interact with each other would remain unclear. Based on symptom network theory, generating symptom networks of multidimensional symptom experiences can provide additional data, such as centrality and density, which have clinical implications and could lead to the development of more precise and individualized interventions.^20^, ^21^ Previous studies have developed symptom networks in various populations, including cancer patients receiving chemotherapy, people living with HIV, and people with mental disorders, to capture complex relationships among symptoms of various chronic diseases.^22^, ^23^, ^24^, ^25^ Symptom networks not only have the function of dimension reduction, through symptom clusters for example, but also can guide health care providers and researchers to identify the core symptoms and to focus on the microlevel interactions among symptoms.^19^

However, few studies have explored the symptom networks of multidimensional symptom experiences in cancer survivors. It remains unclear which symptoms are the core symptoms in long‐term cancer survivors from a perspective that evaluates mechanistic interactions among symptoms. This empirical evidence is needed for the development of personalized and precision symptom management strategies. Therefore, the objectives of this study were to (1) generate symptom networks of multidimensional symptom experiences in cancer survivors and (2) explore centrality indices and density in the symptom networks.

## 2 METHODS

### 2.1 Study design and settings

We conducted a multisite, population‐based cross‐sectional study named the Shanghai CANcer Survivor (SCANS) Report between May and October 2021. Participants were recruited from nine communities in Shanghai, China. As a first‐tier city, Shanghai had more than 450,000 cancer survivors, with all‐cancer mortality of 256 per 100,000 people in 2020. The most common cancers in Shanghai were lung, colorectal, breast, and thyroid cancer. Therefore, the participants in this study are representative of the full population of cancer survivors in Shanghai. The Institutional Review Board of Fudan University School of Nursing approved this study (IRB# TYSQ 2020‐04‐09).

### 2.2 Participants

The inclusion criteria were as follows: (1) Diagnosed with cancer and had completed initial treatment (e.g., surgery, chemotherapy or radiotherapy); (2) aged 18 years or older; (3) provided informed consent. The exclusion criterion was an inability to complete the self‐rating scale due to severe comorbidities and/or cognitive impairment. Finally, a total of 1065 participants were included in the analysis.

### 2.3 Measures

Participants who met the criteria were asked to provide written informed consent before data collection. The survey was delivered by the members of the research team. The following sections were included in the survey.

#### 2.3.1 Self‐reported symptoms

The symptom severity domain of the MDASI was used to assess the prevalence and severity of 13 cancer‐related symptoms (Figure S1).^12^ The participants were asked to rate the severity of symptoms. Symptoms were rated with scores ranging from 0 to 10, with higher scores indicating more severe symptoms. The MDASI, which examines 13 symptom items, has a total score that can range from 0 to 130 in the symptom domain. In our sample, the checklist showed high internal consistency (Cronbach's *α* = 0.970).

#### 2.3.2 Sociodemographic and clinical data

Sociodemographic and health‐related data were collected by a self‐administered questionnaire. The sociodemographic variables included age, sex, ethnicity, marital status, employment status, educational attainment, and primary caregiver. Health‐related variables included type of cancer, stage of cancer, duration of cancer survivorship, types of therapy received, and comorbidities.

### 2.4 Data analysis

#### 2.4.1 Developing contemporaneous symptom networks

All statistical analyses were conducted using R version 1.6.4. The demographic characteristics and severity of symptoms are described using frequencies, percentages, means, and standard deviations. We constructed contemporaneous networks with all 13 symptoms. Each node represented one symptom. Edges in the network represented the conditional independent relationships between two nodes, and the thicker the edges were, the stronger the association between two nodes. We visualized the network using the *qgraph* package. The *spring* layout was used to generate undirected association networks. In this algorithm, nodes with stronger connections are placed closer to each other in the center of the network. A subgroup analysis was performed to identify the difference in networks among populations with various durations of cancer survivorships (<5 years, 5–10 years, and over 10 years). Covariates that were significantly associated with the overall severity were included in the network analysis to identify the real relationships among the 13 symptoms after controlling for confounding factors.

Bootstrapping methods were performed to assess the accuracy and stability of the network by using the R package *bootnet*. Accuracy and stability are two indicators that reflect how accurate and how stable the estimated networks are. The accuracy of the estimated network connections was evaluated by calculating the 95% confidence intervals (CIs) of the edge weight values. We used nonparametric bootstrapping (1000 bootstrap samples) to construct CIs.^26^ The stability was evaluated by calculating the correlation stability coefficient of the expected impact of nodes using a case‐dropping subset bootstrap (1000 bootstrap samples).^27^ The correlation stability coefficient should preferably be >0.5 but at the very least be >0.25.^28^

#### 2.4.2 Node centrality

Node centrality is an indicator for identifying core symptoms from a mechanism perspective. We conducted centrality analysis with the following three centrality indices: Strength, betweenness, and closeness. Symptoms with high values of strength, betweenness, and closeness are regarded as being important from a mechanistic perspective. The strength of a symptom is an indicator of network connectivity. A higher strength centrality means that the symptom is more likely to occur in conjunction with other symptoms. Betweenness is measured by the number of times a node acts as a bridge along the shortest path between two nodes. A node with a higher betweenness centrality has greater network influence. Closeness is indicated by the average distance (inverse distance) between one symptom node and all other nodes. The shorter the path is, the greater the closeness value. ∑_*s*_ (the absolute value of all Spearman coefficients between two nodes) was used as an indicator of network density, which was used in a previous study as an indicator for long‐term prognosis.^22^

#### 2.4.3 Node predictability and difference tests

We used the *mgm* package to identify the predictability for each node. Node predictability is an indicator for assessing how well a given node is predicted by all remaining nodes in a network. A symptom with a high predictability value indicated that we could control the symptom via its neighboring nodes. In contrast, if the predictability value is low, we need to directly intervene in the symptom or look for a marker outside the network.

Finally, we performed a difference test to identify whether the estimations of network connections and centrality for different variables differ. Bootstrapped difference tests were conducted between edge weights and centrality indices in the least absolute shrinkage and selection operator regularization of partial correlation networks based on polychoric correlation matrices.^28^

## 3 RESULTS

### 3.1 Characteristics of participants

This study included 1065 participants in the analysis. The characteristics of the participants are shown in Table 1. Most participants were female (*n* = 712, 66.85%), were of Han ethnicity (*n* = 1062, 99.72%), were married (*n* = 930, 87.32%), had a secondary education level (*n* = 696, 65.35%), and had part‐time jobs (*n* = 733, 68.83%). Breast cancer was the most prevalent cancer among the participants (*n* = 312, 29.30%), followed by gastrointestinal (*n* = 241, 22.63%), head and neck (*n* = 157, 14.74%), and lung cancer (*n* = 154, 14.46%). The average duration of cancer survivorships was 5.46 ± 4.87 years, which ranged from 0.5 to 26.0 years. Regarding the therapies received, most participants received Chinese medicine therapy (*n* = 113, 10.61%), followed by endocrine therapy (*n* = 143, 13.43%), immunotherapy (*n* = 121, 11.36%), and chemotherapy (*n* = 545, 51.17%). The most commonly used therapy was Chinese medicine therapy (*n* = 545, 51.17%), followed by endocrine therapy (*n* = 143, 13.43%) and immunotherapy (*n* = 121, 11.36%). Hypertension was the most prevalent comorbidity among participants, followed by diabetes (194, 18.22%) and cardiovascular disease (*n* = 151, 14.18%).

**TABLE 1:** Characteristics of participants (*n* = 1065)

Characteristics	*n* (%), *M* ± *SD* (IQR)
Age	65.00 ± 11.42 (27–96)
27–40	45 (4.23)
41–65	497 (46.67)
66–80	445 (41.78)
≥81	78 (7.32)
Gender	
Male	353 (33.15)
Female	712 (66.85)
Ethnicity	
Han	1062 (99.72)
Minority	3 (0.28)
Education attainment	
Primary school or below	49 (4.60)
Secondary school	696 (65.35)
Post‐secondary	179 (16.81)
University or above	141 (13.24)
Marital status	
Married	930 (87.32)
Single	135 (12.68)
Employment	
Yes, full‐time job	85 (7.98)
Yes, part‐time job	733 (68.83)
Otherwise	247 (23.19)
Primary caregiver	
Spouse	890 (83.57)
Children	272 (25.54)
Parents	62 (5.82)
Self	17 (1.60)
Type of cancer	
Gastrointestinal cancer	241 (22.63)
Gynecologic cancer	57 (5.35)
Urinary cancer	86 (8.08)
Blood cancer	31 (2.91)
Breast cancer	312 (29.30)
Lung cancer	154 (14.46)
Head and neck	157 (14.74)
Brain cancer	13 (1.22)
Otherwise	34 (3.19)
Stage of cancer	
I	417 (39.15)
II	430 (40.38)
III	113 (10.61)
IV	40 (3.76)
Not clear	65 (6.10)
Duration of cancer survivorships (year)	5.46 ± 4.87 (0.5–26.0)
Types of received therapy	
Surgery	42 (3.94)
Chemotherapy	113 (10.61)
Radiotherapy	29 (2.72)
Endocrine therapy	143 (13.43)
Biological targeted therapy	40 (3.76)
Immunotherapy	121 (11.36)
Chinese medicine therapy	545 (51.17)
Interventional therapy	4 (0.38)
Otherwise	79 (7.42)
Comorbidities	
Hypertension	458 (43.00)
Diabetes	194 (18.22)
Cardiovascular disease	151 (14.18)
Otherwise	49 (4.60)
No comorbidities	496 (46.57)

### 3.2 Symptom prevalence and severity

Table 2 shows the participants' symptom prevalence and severity. Regarding symptom prevalence, fatigue was the most prevalent symptom (*n* = 774, 72.68%), followed by disturbed sleep (*n* = 681, 63.94), difficulty remembering (*n* = 622, 58.40%), lack of appetite (*n* = 556, 52.21%), and emotional distress (*n* = 536, 50.33%). Regarding symptom severity, fatigue was the most severe symptom (2.71 ± 2.67), followed by disturbed sleep (2.34 ± 2.63), difficulty remembering (2.12 ± 2.64), and emotional distress (1.80 ± 2.56). The total severity score in the “less than 5 years cancer survivorship” group was significantly different from that in the two groups with over 5 years of cancer survivorship (*p* = 0.025).

**TABLE 2:** Symptom prevalence and severity of participants (*n* = 1065)

	*n* (%)	*M* ± *SD* (IQR)
Symptom severity domain		
Pain	530 (49.77)	1.71 ± 2.44 (0–10)
Fatigue	774 (72.68)	2.71 ± 2.67 (0–10)
Disturbed sleep	681 (63.94)	2.34 ± 2.63 (0–10)
Distress (emotional)	536 (50.33)	1.80 ± 2.56 (0–10)
Shortness of breath	473 (44.41)	1.49 ± 2.31 (0–10)
Drowsiness	511 (47.98)	1.69 ± 2.47 (0–10)
Dry mouth	479 (44.98)	1.58 ± 2.42 (0–10)
Sadness	457 (42.91)	1.53 ± 2.45 (0–10)
Difficulty remembering	622 (58.40)	2.12 ± 2.64 (0–10)
Numbness or tingling	416 (39.06)	1.41 ± 2.37 (0–10)
Lack of appetite	556 (52.21)	1.71 ± 2.38 (0–10)
Nausea	351 (32.96)	1.17 ± 2.20 (0–10)
Vomiting	296 (27.79)	1.02 ± 2.15 (0–10)
Symptom interference domain		
Relations with others	555 (52.11)	1.82 ± 2.49 (0–10)
Enjoyment of life	586 (55.02)	2.09 ± 2.65 (0–10)
Mood	613 (57.56)	1.91 ± ±2.40 (0–10)
Walking	518 (48.64)	1.91 ± 2.67 (0–10)
General activity	608 (57.09)	1.94 ± 2.48 (0–10)
Working	668 (62.72)	2.27 ± 2.57 (0–10)

### 3.3 Associated factors with the overall symptom severity

Table 3 shows the linear regression models for overall symptom severity. The duration of cancer survivorships (*β* = −0.097, *p* = 0.029), receiving chemotherapy (*β* = 0.198, *p* = <0.0001), receiving Chinese medicine therapy (*β* = −0.111, *p* = 0.008), and having cardiovascular disease (*β* = 0.127, *p* = 0.003) were significantly associated with the overall symptom severity scores. These factors were further included in the network analysis as covariates.

**TABLE 3:** Linear regression model of overall symptom severity

Characteristics	*β*	*p*
Age	−0.051	0.245
Male (compared to female)	−0.022	0.667
Han (compared to minority)	0.010	0.802
Secondary school or below (compared to otherwise)	−0.010	0.812
Married (compared to otherwise)	−0.035	0.392
Type of cancer^a^		
Gastrointestinal cancer	−0.052	0.300
Breast cancer	−0.016	0.781
Lung cancer	0.091	0.055
Head and neck	−0.050	0.300
Stage of cancer	0.056	0.183
Duration of cancer survivorships	−0.097	0.029
Types of received therapy^a^		
Chemotherapy	0.198	<0.0001
Endocrine therapy	−0.006	0.897
Immunotherapy	0.059	0.154
Chinese medicine therapy	−0.111	0.008
Comorbidities^a^		
Hypertension	0.077	0.072
Diabetes	−0.008	0.845
Cardiovascular disease	0.127	0.003

*Note*: $R_{\mathrm{adj}}^{2}$ = 0.106, *F* = 3.621, *p* = 0.000.

^a^ To ensure the statistical power, only covariate with >100 sample size was included.

### 3.4 Density, accuracy, and stability of symptom networks

**FIGURE 1:**
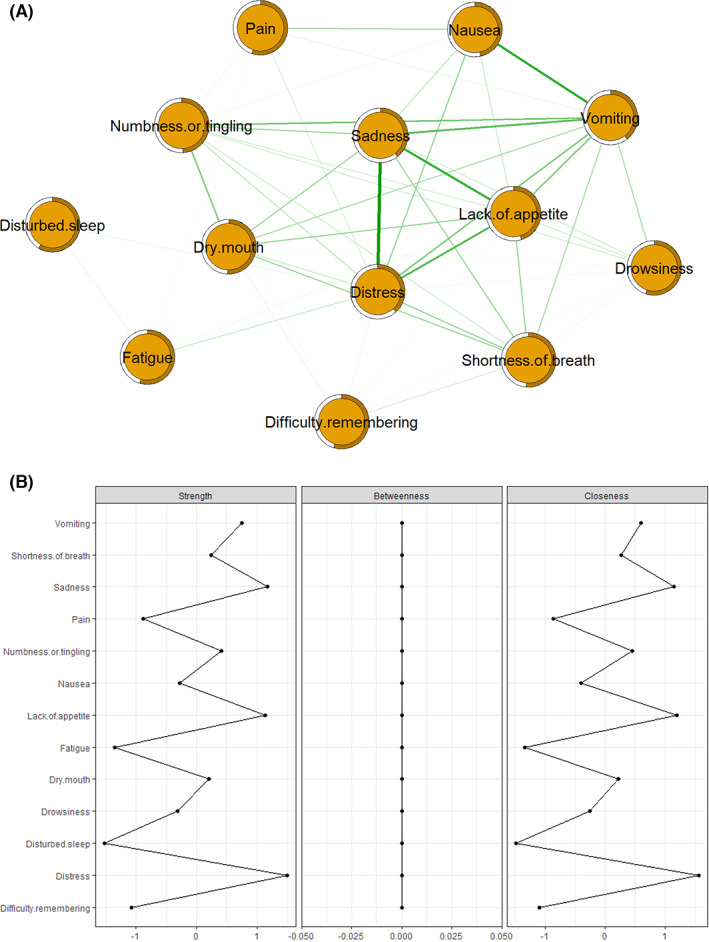
Symptom networks and centrality measures in the full sample network (*N* = 1065). (A) Symptom network and predictability of 13 symptoms; (B) strength, betweenness, and closeness of 13 symptoms.

**FIGURE 2:**
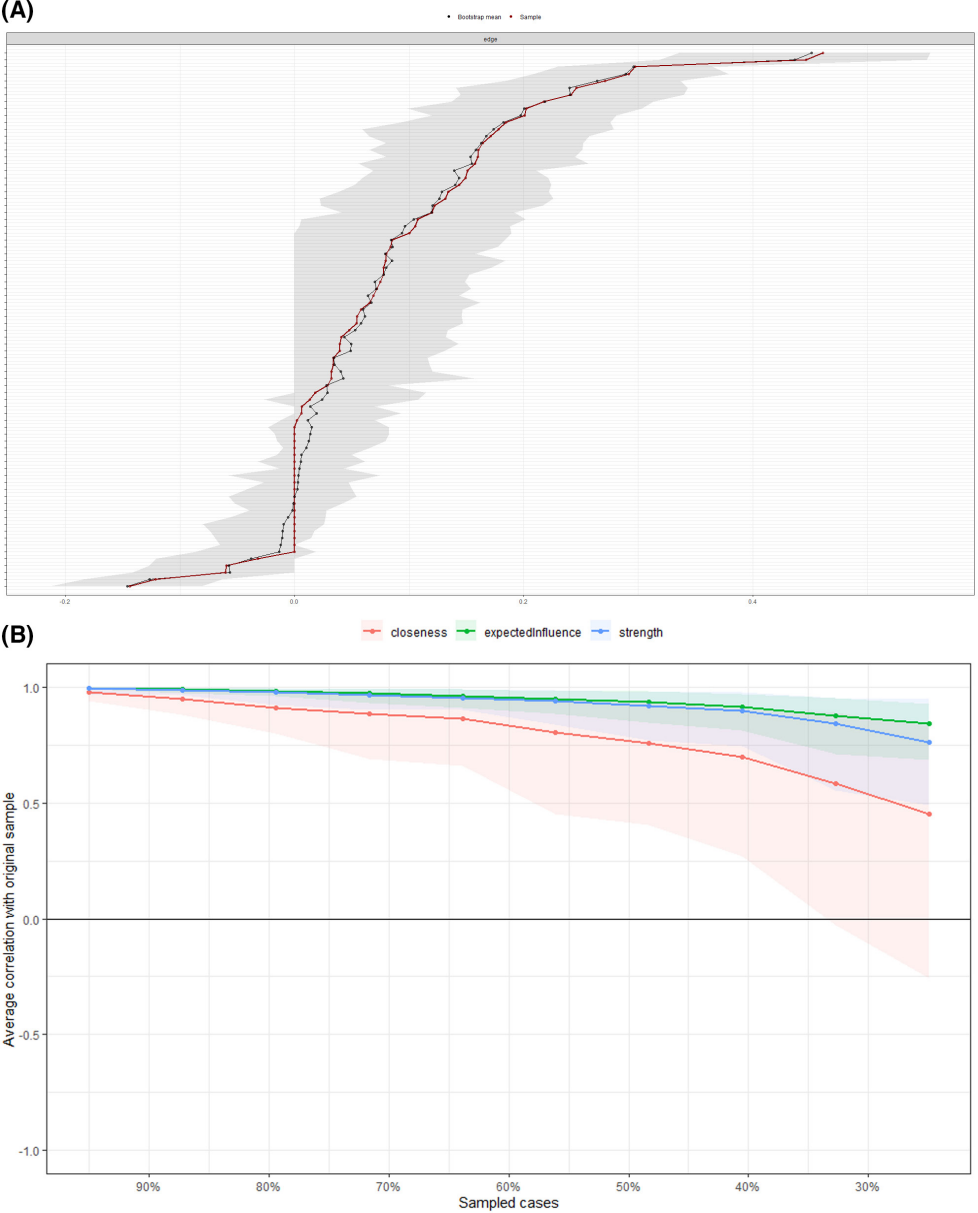
Accuracy and stability of the symptom networks. (A) Bootstrap analysis results of the edge weights; (B) correlation stability coefficient for strength, expected influence, and closeness.

Figure 1A shows the symptom network of the multidimensional symptom experiences in cancer survivors. The density of the full sample network is 111.56. Figure 2A shows the bootstrap analysis results of the edge weights. The bootstrapped CIs were small, which showed good accuracy of the network. For the bootstrap subset (Figure 2B), the correlation stability coefficient was 0.75 for expected influence and 0.59 for strength, suggesting that the network remained stable. In the subgroup analysis (Figures S2–S10), the density of the “less than 5 years” networks was significantly different from that of the “5–10 years” and “over 10 years” networks (*p* < 0.001). The accuracy and stability of the subgroup networks are shown in supplementary files.

### 3.5 Centrality, predictability, and difference test

**FIGURE 3:**
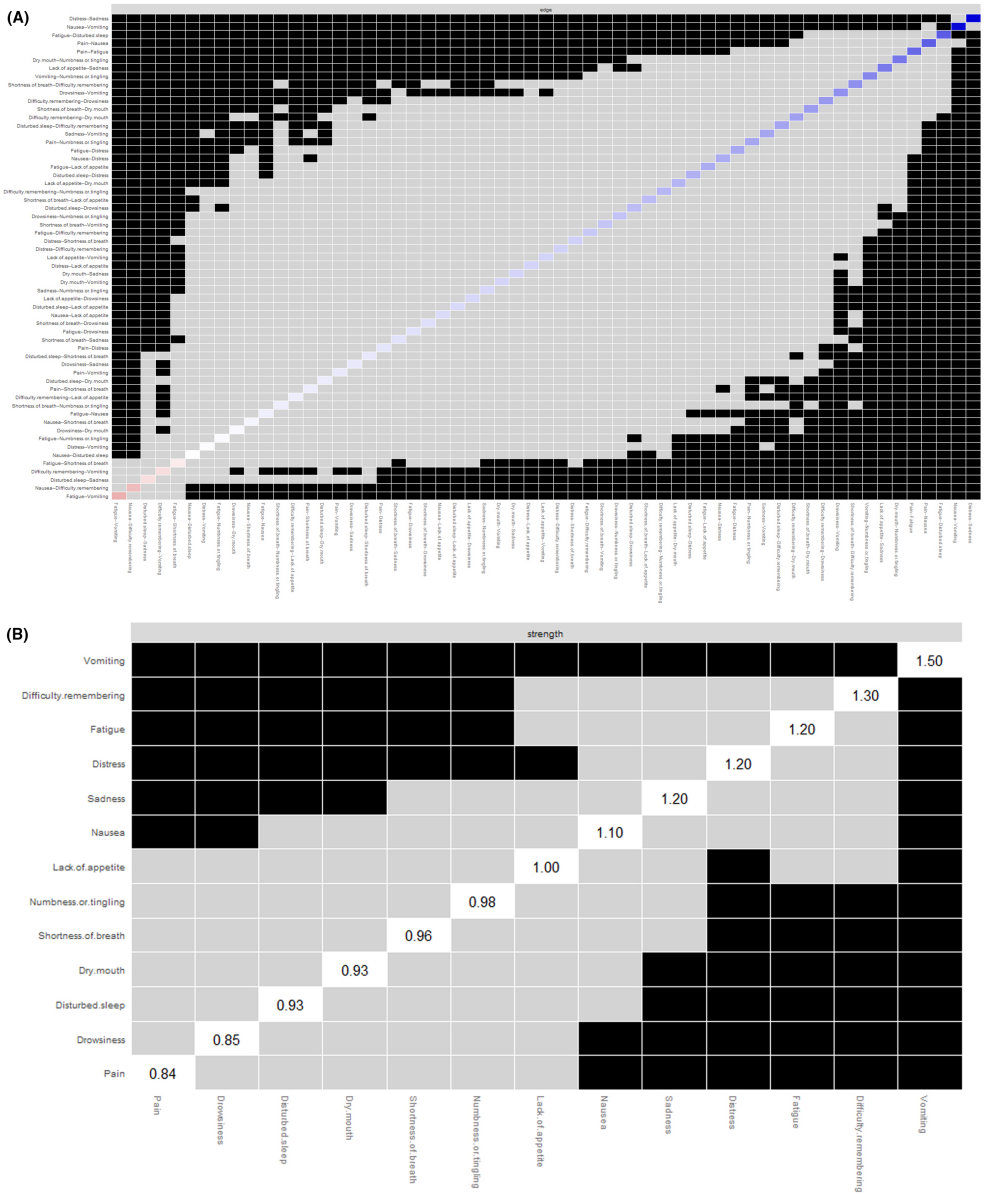
Results of difference tests. (A) Bootstrapped difference test for edges; (B) bootstrapped difference test for nodes.

Figure 1B shows two centrality indices: Strength and closeness. Distress (*r*_*s*_ = 9.18, *r*_*c*_ = 0.06), sadness (*r*_*s*_ = 9.05, *r*_*c*_ = 0.06), and lack of appetite (*r*_*s*_ = 9.04, *r*_*c*_ = 0.06) had the largest values for strength and closeness. Fatigue (*r*_*s*_ = 8.04, *r*_*c*_ = 0.06) had lower centrality than other symptoms, with the exception of disturbed sleep (*r*_*s*_ = 7.97, *r*_*c*_ = 0.06). Predictability is presented as circles around the nodes in Figure 1A. The node predictability values ranged from 38.5% to 58.6%. Disturbed sleep, pain, and drowsiness had the highest predictability, showing that 58.6%, 55.2%, and 55.1% of their variance can be explained by their neighboring symptoms. Figure 3A shows the results of the bootstrapped edge difference test. The bootstrapped difference test for edge weights showed that the two strongest edge weights, “distress and sadness” and “nausea and vomiting”, were significantly different from approximately 95% of the other edge weights. Figure 3B shows the results of the bootstrapped node difference test. Vomiting significantly differed from other nodes (DT_*s*_ = 1.50).

## 4 DISCUSSION

This is the first study that explored the symptom network of multidimensional symptom experiences in cancer survivors. Contemporaneous symptom networks can help researchers identify the most important symptom in a network structure and support health care providers and researchers in designing precisely personalized therapies. In this study, we found that while fatigue was the most severe symptom in cancer survivors, the centrality of fatigue was lower than that of the majority of other symptoms. Distress, sadness, and lack of appetite were the core symptoms in cancer survivors, especially in survivors with <5 years of survivorship. The density of networks in survivors with <5 years survivorship was significantly higher than that in survivors with >5 years, which indicated that the long‐term prognosis of cancer therapy from a symptom perspective may differ between survivors with less than and those with more 5‐year survivorships.

Fatigue has been a widely studied symptom in cancer survivors across various contexts in previous studies. These studies have identified fatigue as the most prevalent, severe, and distressful symptom in survivors with various types of cancer.^29^, ^30^ The overall prevalence of fatigue was 49%, ranging from 26.2% to 60.6% based on the results of 129 studies.^31^ However, our study found that the centrality indices of fatigue, including strength and closeness, had the second smallest values compared to other symptoms.

Our results indicated that fatigue may not be the central symptom in cancer survivors despite its high prevalence and severity. Based on symptom prevalence and severity, it remains difficult to understand the mechanisms underlying symptom deterioration and alleviation. Network centrality provides a symptom‐level indicator that enables researchers to identify potentially more central symptoms from a mechanistic perspective.^28^ Node centrality is an indicator for identifying core symptoms from a mechanism perspective. Based on the symptom network, fatigue was regarded as a sentinel symptom in previous studies due to its high level of centrality. Data from the “Paradigm Shift in Chemotherapy‐Induced Nausea and Vomiting” study showed that in 209 cancer survivors receiving adjuvant chemotherapy, fatigue was identified as the sentinel symptom in the first and second cycle of chemotherapy.^32^ However, in their recent study, Rha and Lee analyzed longitudinal data from 249 patients with cancer receiving chemotherapy and found that the centrality of fatigue decreased after the fourth cycle of chemotherapy.^33^ They indicated that the core position of fatigue in symptom networks may have been due to the use of chemotherapy and durations of cancer survivorships. Our study echoed Rha and Lee's findings and further showed that the centrality of fatigue was even lower in a population with over 5 years of survivorship. Cancer therapies, including chemotherapy, radiation therapy, immunotherapy, and surgery, may contribute to high levels of the centrality of fatigue in symptom networks.^24^ The duration of cancer survivorships in our sample was over 5 years, and the majority of participants received Chinese medicine therapy, which has been regarded as having few side effects. These two reasons may contribute to fatigue being low centrality in our study.

This study also found that distress and sadness were the core symptoms in cancer survivors, which indicated that for long‐term cancer survivors, early detection and psychological adjustments that relieve emotional distress should be crucial components of long‐term management. Previous studies have reported that long‐term cancer survivors face a series of psychological distresses, including powerlessness, insecurity, social disconnection, and loneliness and have difficulties understanding and managing their emotions.^34^, ^35^, ^36^ Psychological distress might come from financial and insurance issues, delayed physical effects that emerge many years after the completion of therapy, social adaptation, and fear of reoccurrence.^37^, ^38^, ^39^ From the results of a national survey by Hoffman et al., the prevalence of psychological distress and negative emotions was reported to be higher in long‐term survivors than in populations who were never diagnosed with cancer.^40^ It is crucial to address psychological distress not only because this would maintain high levels of quality of life but also because these symptoms may hamper survivors' health screening behaviors, which could detrimentally affect other symptoms.^37^, ^41^

Emotional distress plays a central role in cancer survivors at all durations of survivorship. When survivors live longer, they may face different struggles at different stages of survivorship from survival crisis to back‐to‐work anxiety. It is warranted that future studies examine the centrality of psychological distress in the symptom network in longitudinal data. Emotional distress is an overall state of mental suffering that includes a wide variety of forms such as anxiety, depression, worrying, and anger.^42^ Health care providers need to explore the causes and psychological symptoms of emotional distress. Mindfulness‐based interventions, meaning‐enhancing interventions, dignity therapy, life review, and spiritually‐focused meditation were proven to be effective in ameliorating emotional distress for cancer survivors.^43^, ^44^ These psychosocial therapies should be tailored to the individual specific requirements of cancer survivors, whether the aim is to manage psychological symptoms or enhance the quality of life.

In addition, we found that the network density in survivors with <5 years of survivorship was significantly higher than that in survivors with >5 years after controlling for all covariates, indicating that the long‐term prognosis of cancer therapy in the >5 years groups was significantly better than that in the <5 years group. Although the severity between the <5‐year group and the >5‐year groups was also significantly different, the network density showed a greater difference between the two groups than using severity as an indicator. This result was in line with previous studies showing that network density could be regarded as a more sensitive indicator than severity to distinguish populations in various courses of disease.^22^, ^41^ Zhu and colleagues conducted a cross‐sectional study with 2927 people living with HIV and found that while participants had the same levels of symptom severity, individuals with a denser network perceived poorer health conditions.^45^ Schweren and colleagues' cohort study was conducted with 465 adolescents with depression and identified denser networks as a behavioral indicator that predicted longer illness duration.^46^ Although these two studies were conducted in populations other than cancer survivors, the extension of the conclusions regarding network density from other populations to the population at large was acceptable. We found that network density could indicate the long‐term prognosis of cancer therapy from a symptom perspective. We suggest that cohort studies should be carried out and that longer follow‐ups (more than 5 years) are warranted to evaluate the trajectory of network density in cancer survivors. It is also recommended to further analyze the relationships between symptom density and quality of life in cancer survivors.

### 4.1 Limitations

Despite the many strengths of our study, it has several limitations. First, the cross‐sectional design and convenience sampling method limit the generalizability of the findings. Our study was not able to determine causality among symptoms. Longitudinal studies are warranted to examine dynamic networks to identify which symptoms detrimentally affect other symptoms. Second, we did not include participants who could not complete the self‐rating scale due to severe comorbidities and/or cognitive impairment. Therefore, the severity and centrality of symptoms may have been underestimated. Finally, survivorship bias among the participants may have existed, and the effect sizes of the prevalence, symptom severity, centrality indices, and network density may have been underestimated, especially in the group with >5‐year survivorship.

## 5 CONCLUSION

The findings of our study identified symptom networks of multidimensional symptom experiences in 1065 cancer survivors. Our study demonstrates the need for the assessment of centrality indices and network density as an essential component of cancer care. We recommend evaluating symptom severity based on real‐world clinical follow‐up data to generate symptom networks and centrality indices that could optimize symptom management strategies. Future studies are warranted to develop dynamic symptom networks and follow the trajectories of centrality indices in longitudinal data to explore the causality among symptoms and markers of interventions.

## AUTHOR CONTRIBUTIONS

Jing Zhu and Weijie Xing conceptualized and supervised the study. Yanling Sun, Yi Kuang, Xiaoyi Yuan, and Haiyan Gu collected data. Zheng Zhu and Weijie Xing performed data analysis. Zheng Zhu and Weijie Xing wrote the manuscript. All authors provided critical feedback.

## FUNDING INFORMATION

This work was supported by the National Natural Science Foundation of China (Grant Number: 72004034) and China Medical Board Open Competition Program (Grant Number: 20–371).

## CONFLICT OF INTEREST

All authors declare no disclosures.

## ETHICS APPROVAL STATEMENT

The Institutional Review Board of Fudan University School of Nursing approved this study (IRB# TYSQ 2020‐04‐09).

## PATIENT CONSENT STATEMENT

Participants who met the criteria were asked to provide written informed consent before data collection.

## PERMISSION TO REPRODUCE MATERIAL FROM OTHER SOURCES

NA.

## CLINICAL TRIAL REGISTRATION

NA.

## Supporting information


Figure S1
Figure S2
Figure S3
Figure S4
Figure S5
Figure S6
Figure S7
Figure S8
Figure S9
Figure S10

## Data Availability

The data that support the findings of this study are available from the corresponding author upon reasonable request.
