# Supplementary material for: Contemporaneous symptom networks of multidimensional symptom experiences in cancer survivors: A network analysis
Source: Cancer Med. 2022 Jun 1;12(1):663–73. doi: 10.1002/cam4.4904 (PMC9844664; doi:10.1002/cam4.4904)
Supplement: Supplementary file 1 — Figure S1 Figure S2 Figure S3 Figure S4 Figure S5 Figure S6 Figure S7 Figure S8 Figure S9 Figure S10 [file CAM4-12-663-s001.docx]

**Supplement file**

**
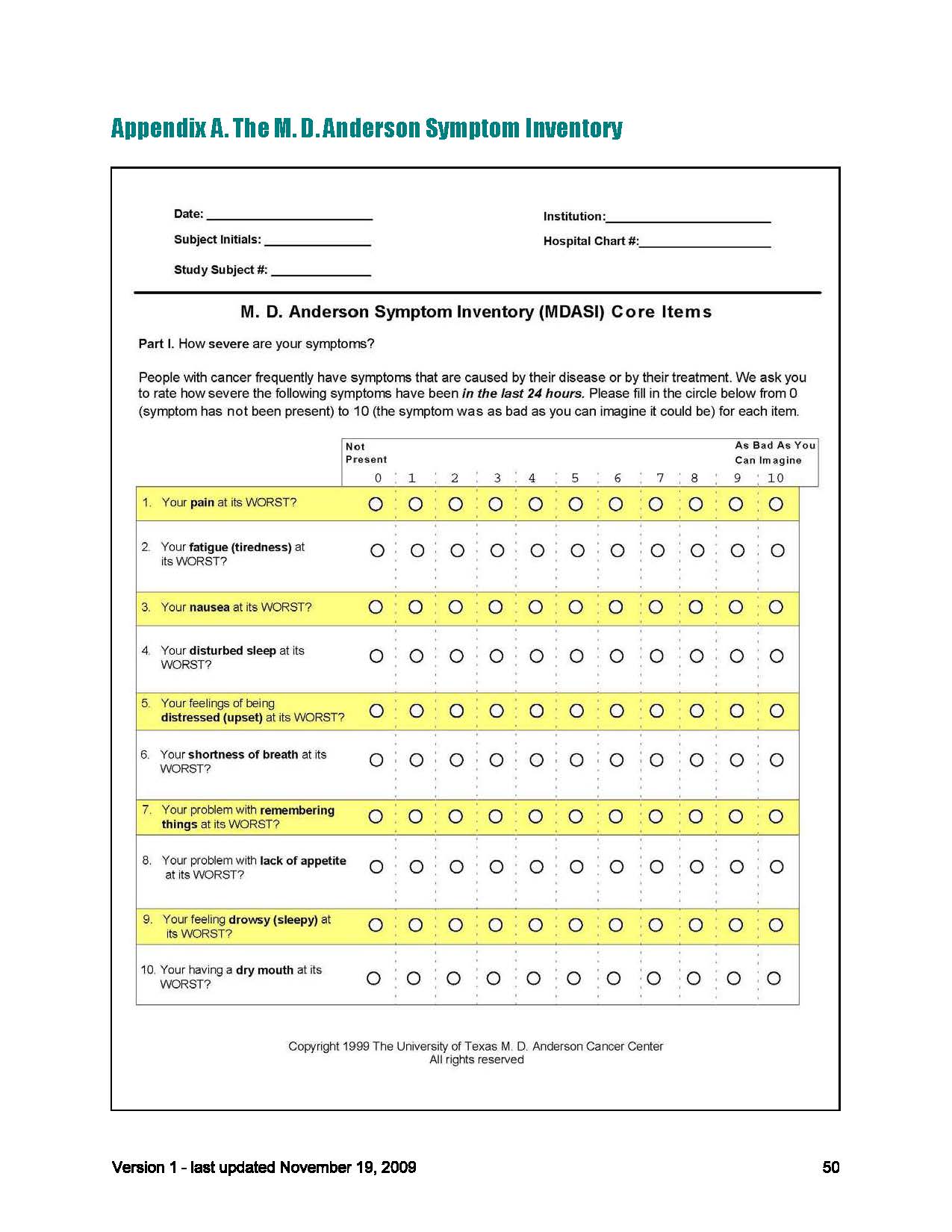
**

**
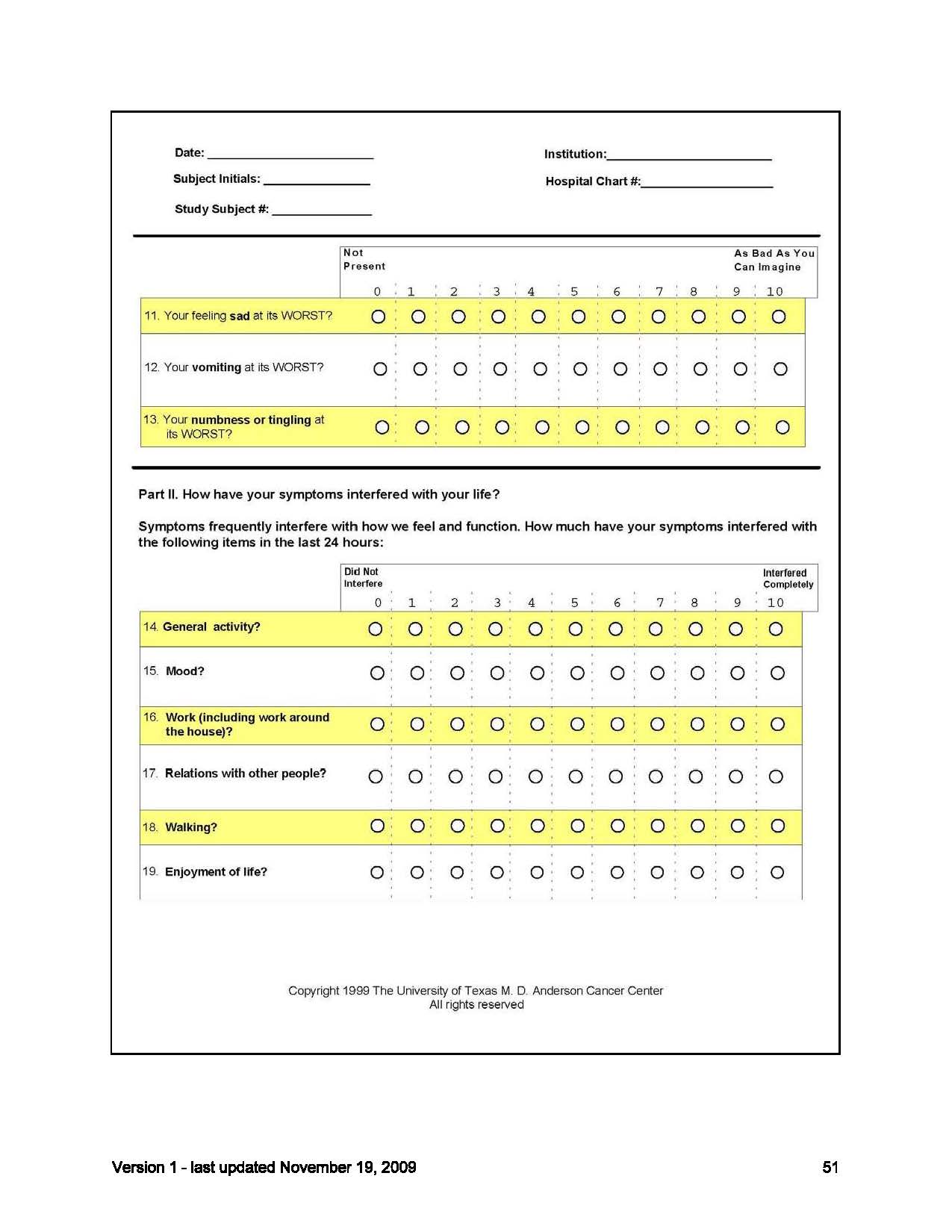
**

**eFigure 1** MD Anderson Symptom Inventory (MDASI)-symptom severity section

**
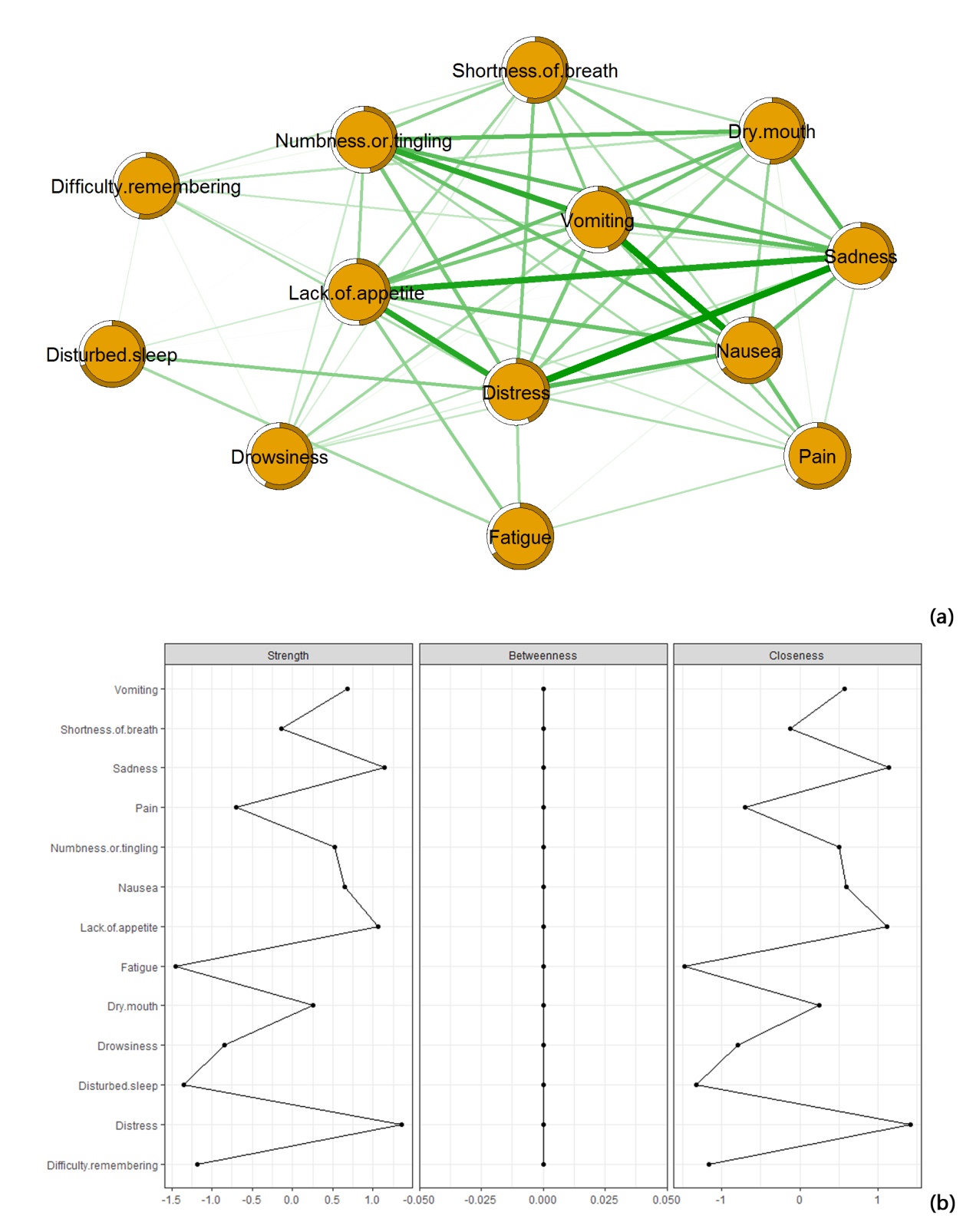
**

**eFigure 2** Symptom networks and centrality measures in the cancer survivorships with less than 5 years. (a) Symptom network and predictability of 13 symptoms; (b) Strength, betweenness, and closeness of 13 symptoms.


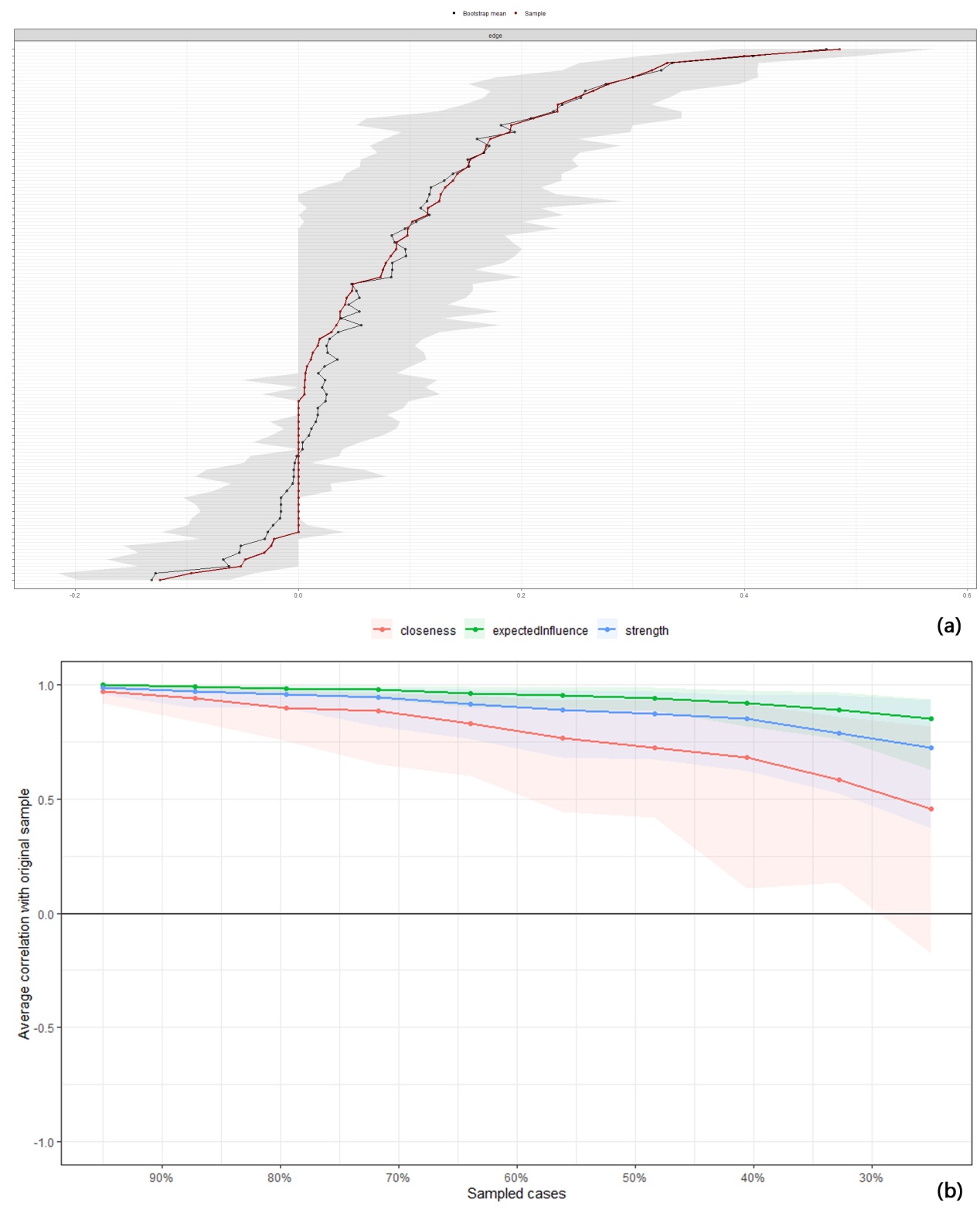


**eFigure 3** Accuracy and stability of the symptom network in the cancer survivorships with less than 5 years. (a) Bootstrap analyses results of the edge weights; (b) correlation stability coefficient for strength, expected influence, and closeness.

**
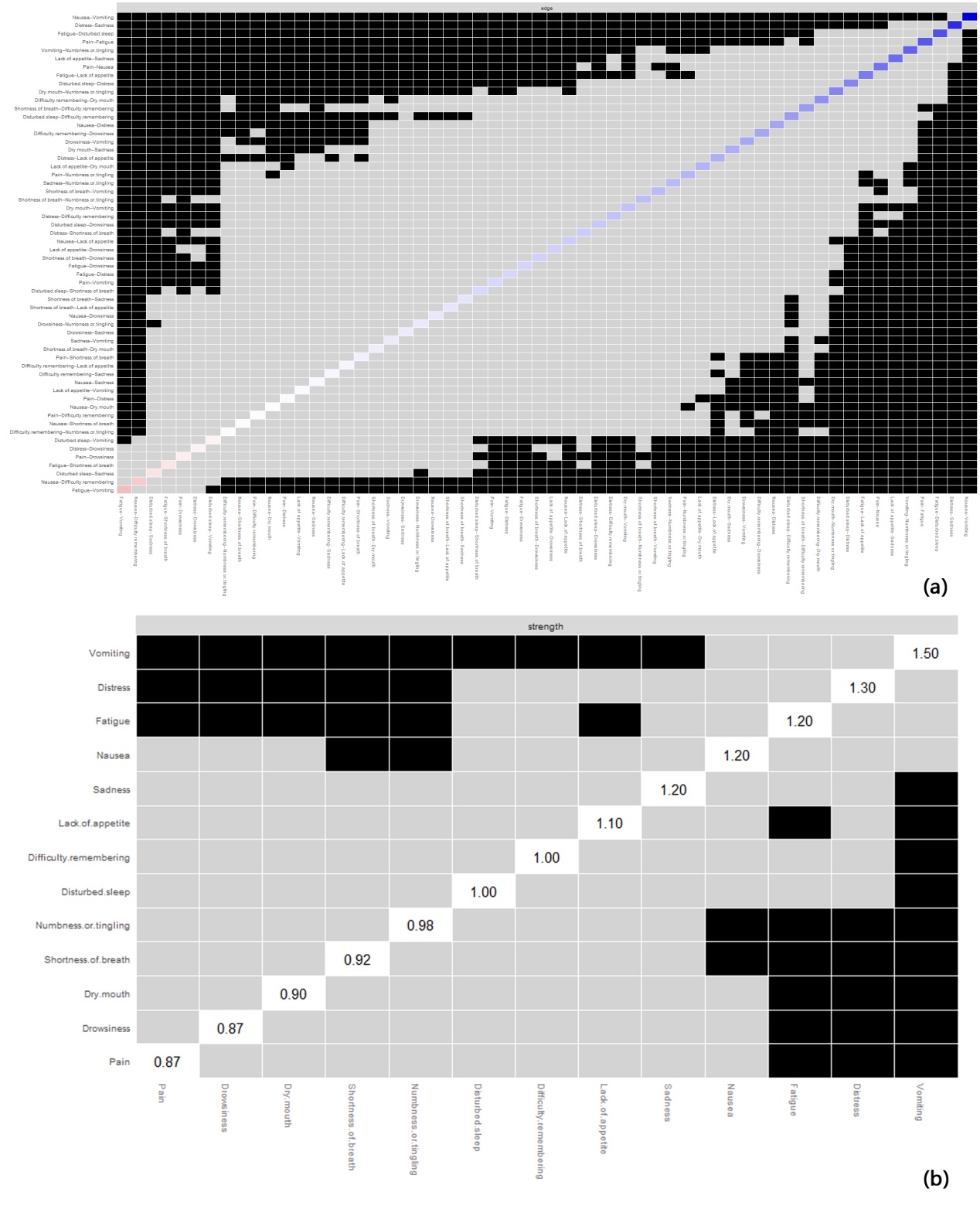
**

**eFigure 4** Results of difference tests in the cancer survivorships with less than 5 years. (a) Bootstrapped difference test for edges; (b) bootstrapped difference test for nodes.


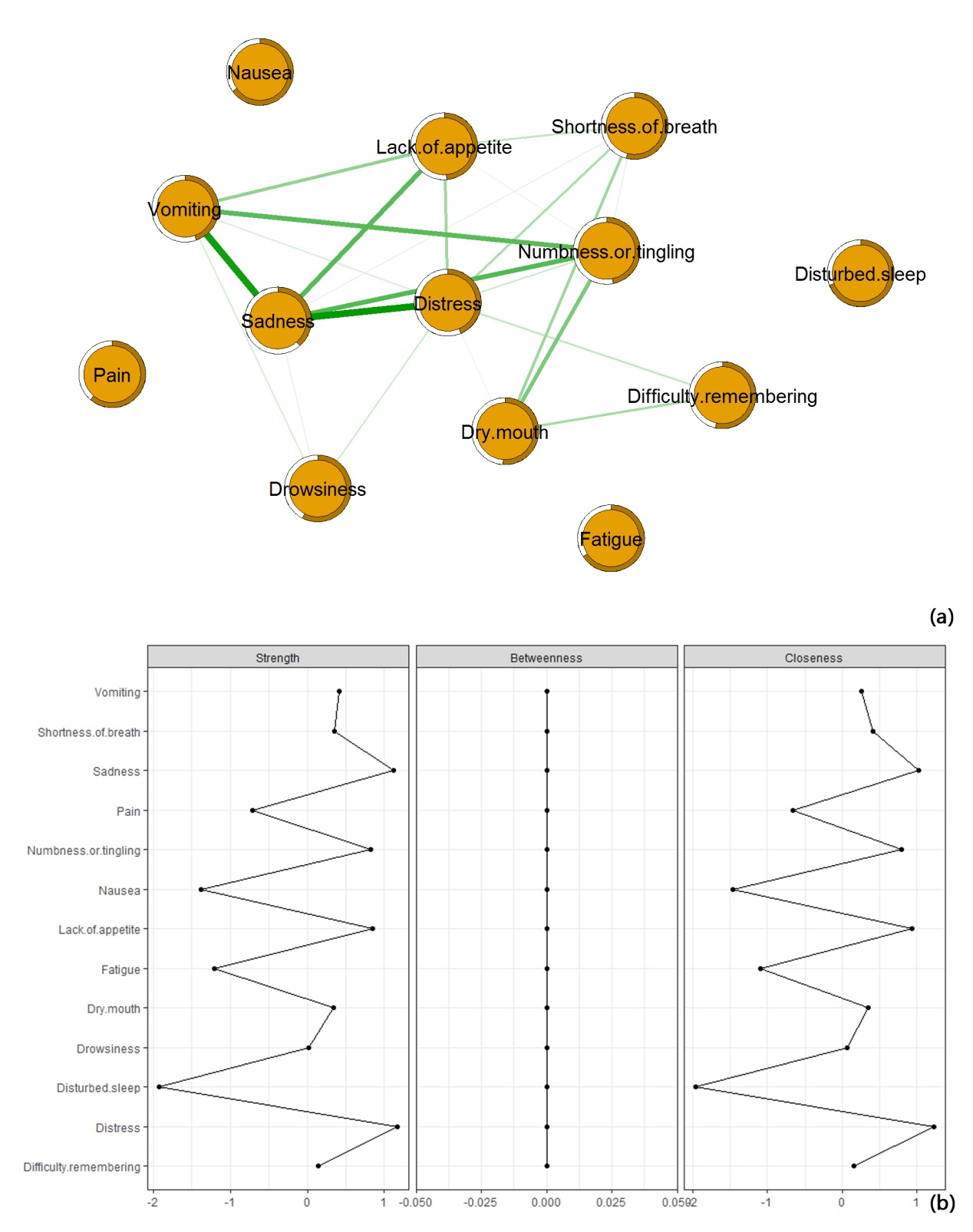


**eFigure 5** Symptom networks and centrality measures in the cancer survivorships with 5-10 years. (a) Symptom network and predictability of 13 symptoms; (b) Strength, betweenness, and closeness of 13 symptoms.


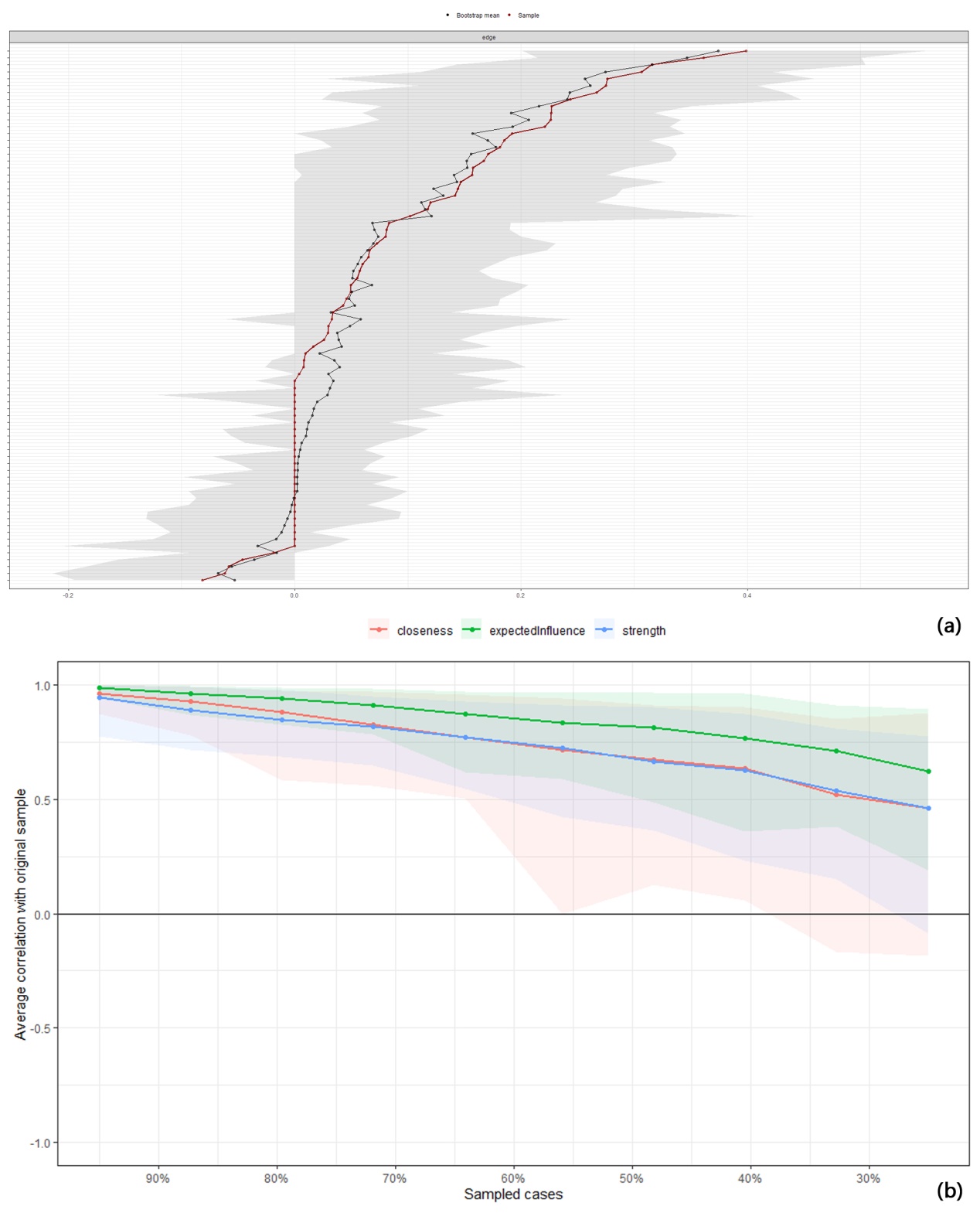


**eFigure 6** Accuracy and stability of the symptom network in the cancer survivorships with 5-10 years. (a) Bootstrap analyses results of the edge weights; (b) correlation stability coefficient for strength, expected influence, and closeness.


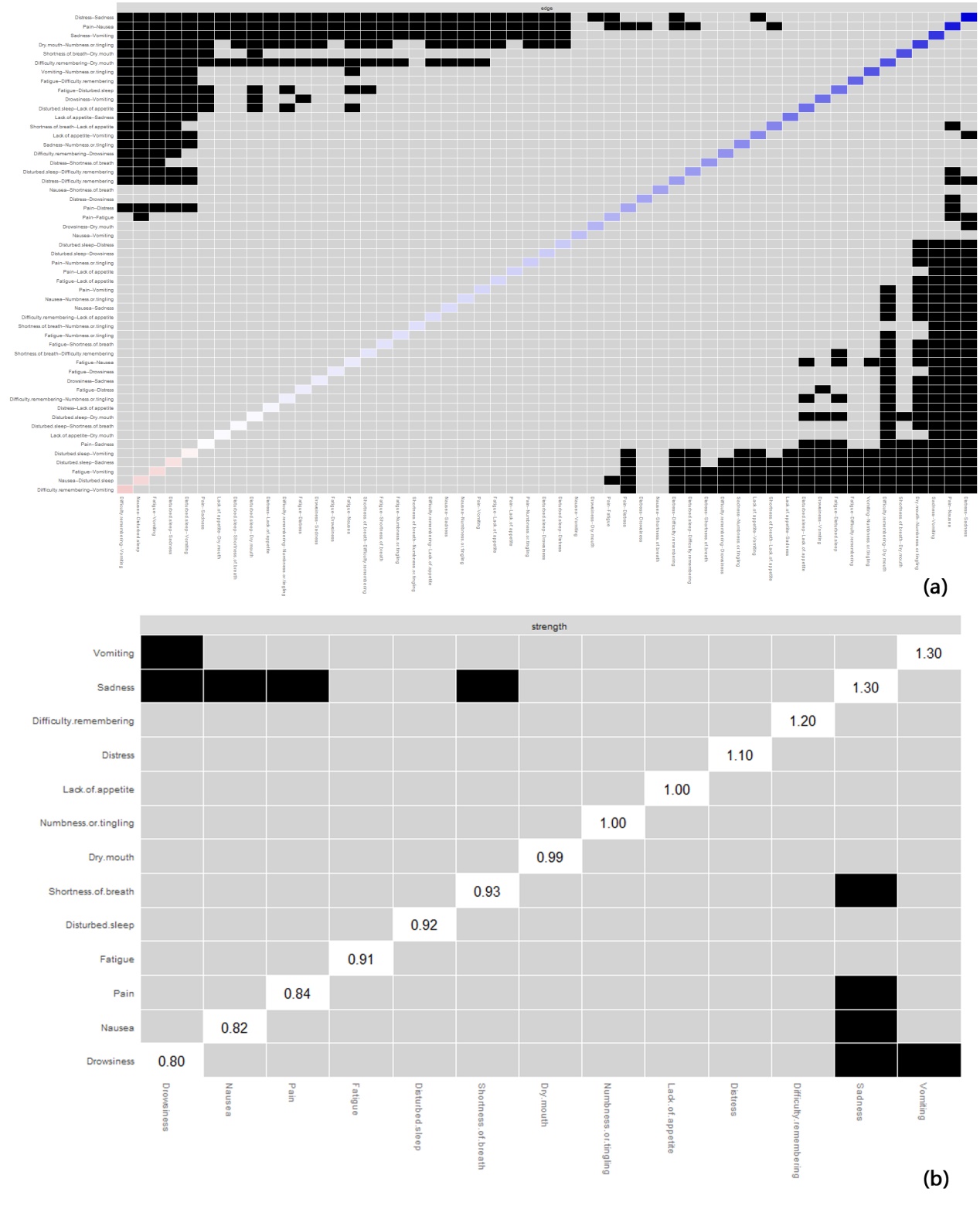


**eFigure 7** Results of difference tests in the cancer survivorships with 5-10 years. (a) Bootstrapped difference test for edges; (b) bootstrapped difference test for nodes.


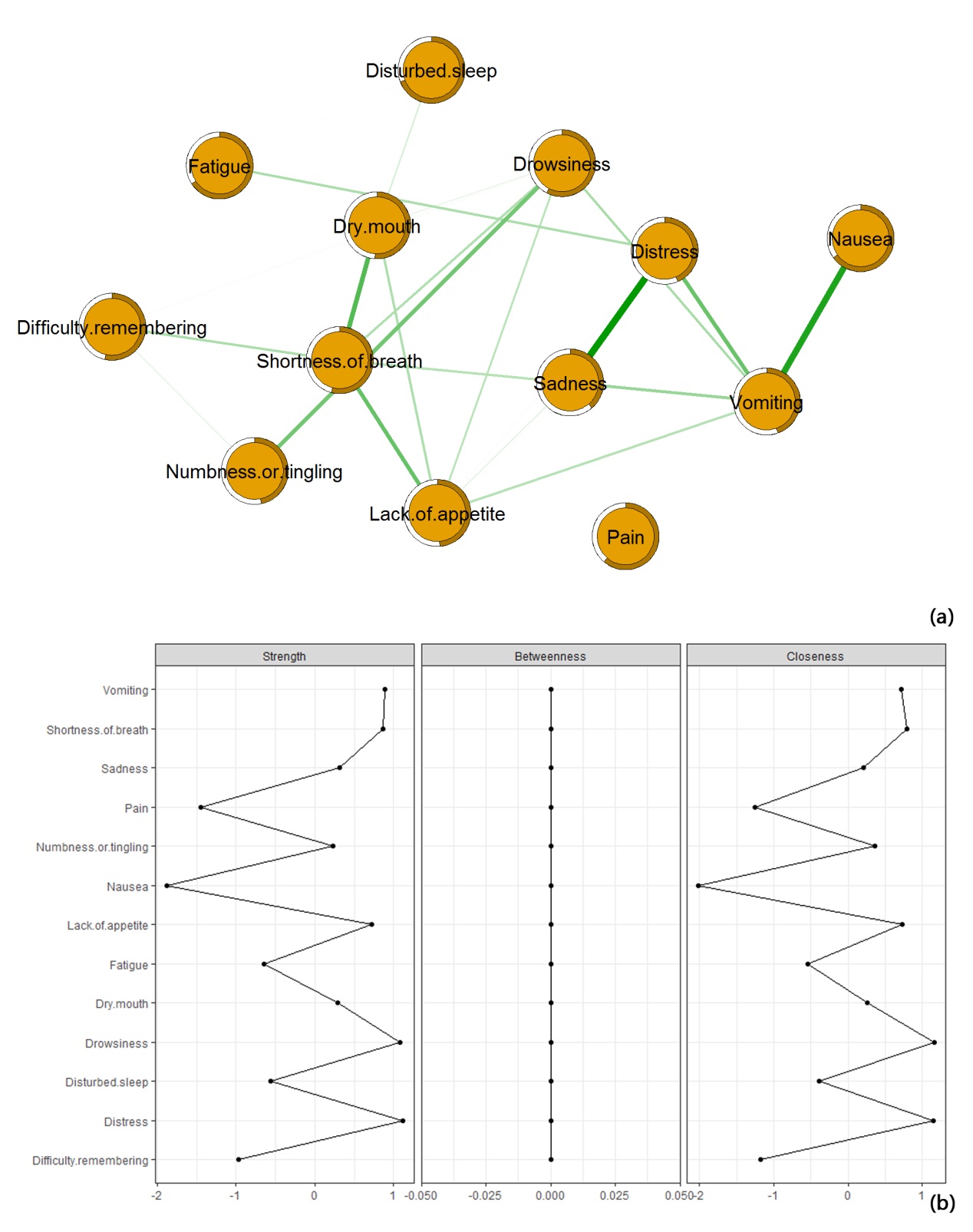


**eFigure 8** Symptom networks and centrality measures in the cancer survivorships with over 10 years. (a) Symptom network and predictability of 13 symptoms; (b) Strength, betweenness, and closeness of 13 symptoms.

**
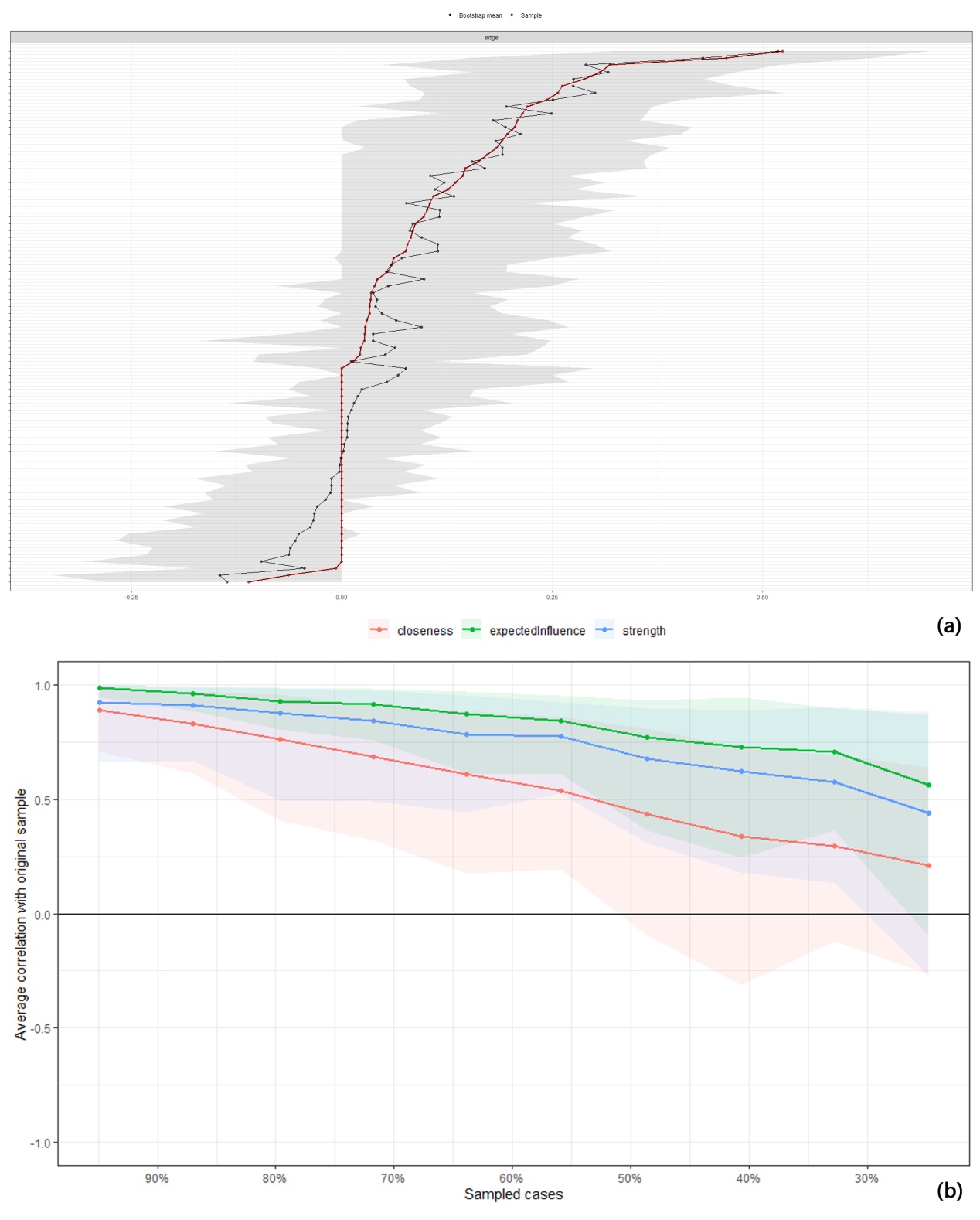
**

**eFigure 9** Accuracy and stability of the symptom network in the cancer survivorships with over 10 years. (a) Bootstrap analyses results of the edge weights; (b) correlation stability coefficient for strength, expected influence, and closeness.


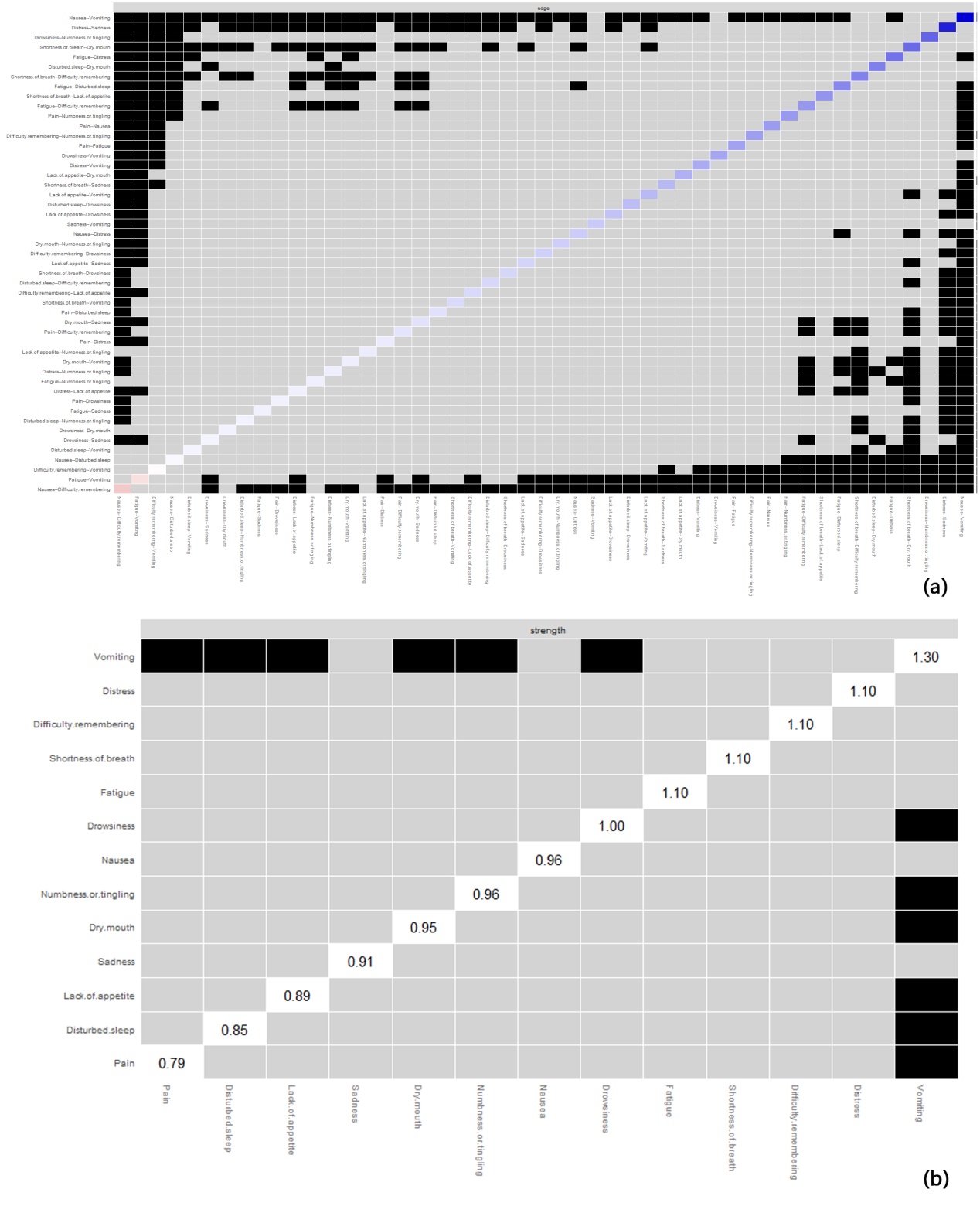


**eFigure 10** Results of difference tests in the cancer survivorships with over 10 years. (a) Bootstrapped difference test for edges; (b) bootstrapped difference test for nodes.
